# Chloroform; Its Uses, Etc.

**Published:** 1855-04

**Authors:** P. A. Allaire

**Affiliations:** Aurora


ART. III.—*Chloroform:* its Uses, etc. By P. A. ALLAIRE, M.D.

THIS powerful agent is now in the hands of nearly every physician, but its numerous uses are not yet well understood, nor have I seen a good resume of its properties and effects. Chloroform is used in three methods:

I. By inhalation.

II. By the stomach; and

III. Locally.

Its effects when used by inhalation are:

1. Anæsthetic, relieving present and preventing anticipated pain.

2. Relaxation of Tissues.

3. Production of Sleep.

4. To prevent and relieve spasm and convulsion.

5. To procure perfect quiet, necessary in some operations.

6. As an anti-emetic.

1. To produce anæsthesia is the most common reason of the use of Chloroform in practice; with this view it is much used for the relief of Neuralgia, and some cases are recorded of cures from it in this affection, but its curative effect here is more frequently secured when given by the mouth, and it is often of much efficacy when locally applied. In all the more painful operations, the Anæsthetic effect of Chloroform is now generally had recourse to, especially where "shock" is feared. This depressing effect on the nervous system is in a great degree prevented, and surgeons have justly and generally accepted Chloroform in these cases as a great boon; it also in many instances of severe operative proceedure leaves the patient in that quiet placid condition which so greatly tends to secure natural sleep, and every surgeon knows the importance of this state as tending to promote a good recovery.

In the minor operations it should never be had recourse to, the pain and shock being slight, there is no propriety in incurring any risk. The only case in which death followed the inhalation of Chloroform in this part of our country, was, where it was given to prevent pain on the removal of part of a finger. Its use is also now common in labor, but it should be used here only when this process is unusually painful or where some operation is to be performed. In parturition where relief of excessive pain is the object, Chloroform does not require to be given in such large doses as in surgical practice, the intention here being to keep the patient in a semi-conscious state, the uterine contractions being in this condition unaffected. When the handkerchief is used an ounce an hour is usually sufficient, a smaller quantity is oftener required than a larger. If necessary the woman may be kept in this way for hours together with safety, where the agent is good, is carefully used, is not improper from local disease or idiosyncracy and she is allowed to come from under its influence every hour. This is necessary that we may know that all is going on well.

If an operation is to be performed on the parturient female, the Anæsthetic as a general rule should not be given, except in turning, and after labor, in extraction of the placenta, but if necessary in instrumental delivery, as it may be from great nervous irritability, timidity, etc.; then a perfectly passive and apathetic state should be induced, before an instrument is introduced, and this should be kept up until they are removed, for the very evident reason that unless the woman is perfectly insensible there may be involuntary motion, and each movement of her pelvis while instruments are within it endangers the injury of the contained organs, perfect quiet is therefore all-important. When the hand only is present the sleep may be less perfect. The safe way is in these operations never to give chloroform. The careful man in operating always wishes to ask frequently the question, "Madam, do I hurt you."

2. Relaxation of tissues is perhaps more thoroughly effected by Chloroform when inhaled than by any other known agent. The warm bath, opium, tartar emetic, vensection, &c., should however always be used in preference when they will effect our purpose, as being safer in our present knowledge. But when they fail as they often will, or when we are well assured they cannot succeed, then we may rely with much confidence on the relaxation produced by Chloroform. We must however in these cases produce the full effect of the medicine. Dislocation of the femur, compound fracture, and to promote the taxis in hernia, are the chief if not the only known cases in which it is necessary to use it for this purpose.—Many cases are now on record of hernia reduced, when an operation would doubtless have been performed had not this agent been known; its action here is not only by the relaxation produced, but also by preventing the pain induced by the necessary manipulation and that vast involuntary muscular resistance which is always made under these circumstances.

3. In the production of sleep, Chloroform is often an admirable agent; many cases of Delirium Tremens have been successfully treated by it, and that restless, sleepless state which often occurs in Typhus and Typhoid fevers, and which always ends fatally if sleep is not soon had, may be frequently relieved by the inhalation of Chloroform. I however generally prefer in these circumstances to give it by the stomach because where Hyperæsthenia Cerebri is present, there is always much exhaustion and depression of the nervous system, and as Chloroform is a powerful sedative when given by inhalation, it will not be found so safe as when taken in the stomach, for in this mode of exhibiting it, it has a very slight stimulating effect, and in these peculiar cases a very excellent soporific influence; but remedies in delirium and especially in Delirium Tremens cannot always be given by the mouth in consequence of violent resistance by the patient, then Chloroform by inhalation if carefully watched and perseveringly used, frequently leads to the best results. It should not here be carried to its full extent: the moment quiet is induced it should be suspended, and the opportunity seized for the administration of stimulants or other needed remedies, the Chloroform being resumed

20

again before its effects have entirely subsided; generally it will be found most useful in those instances which are usually called "sthenic" and which are the very ones least likely to be benefitted by the usual opiate treatment.

4 To prevent and relieve spasm and convulsion. Chloroform has been used successfully in etanus, traumatic and idiopathic.—It should not be carried to its full extent if less will effect our purpose, as in all cases of tonic spasm (not local) the nervous system is rapidly exhausted. Might not its administration by the stomach in large doses here be useful? In hydrophobia it is the only effectual agent we have to smooth the passage to the grave.

A considerable number of cases of chorea, asthma, colic, dysmenorrhoea, hiccup, hysteria, and some anomalous forms of spasm are reported as being benefitted and some cured by the inhalation of Chloroform. The relation of a single case will perhaps best illustrate the use of the remedy in this class of affections:

CASE.—Was called to Mrs. —— on the evening of July 30, 1853. Is now nearly six months in pregnancy; a robust, healthy woman, aet. 38; has had six or seven miscarriages in the last six years, always about the sixth month. When I have attended her on these occasions the fœtus and placenta have been of a perfectly normal appearance, and she has had no apparent uterine disease in the intervals. She has heretofore usually been treated with bleeding, opiates, and rest. Her symptoms are the same now as they have uniformly been in former miscarriages.

*Present Condition.*—Acute persistent, lancinating pain in the right side of the uterus, a little tenderness on pressure, pain increased on motion, cannot lie down, diarrhœa, pulse full—80, tongue natural, appetite good, no expulsive effort or hemorrhage, has been in this condition thirty-six hours. Treatment.—Venesection, ℥xv.

℞ Chloroform.

Ol. Olive.

Of each equal parts—Mix.

The liniment to be freely rubbed over the seat of pain every hour and then a flannel cloth saturated with the liniment to be laid on and covered with oil silk; if sleep is not obtained in two hours then to inhale from the bottle of liniment till perfectly relieved.

July 31, Morning—Had some relief from bleeding and liniment, but could not rest; then inhaled from the bottle and had some sleep; looks rested and more cheerful; diarrhœa better; still some pain; has passed the night in easy chair. To restrict diet and continue chloroform locally and by inhalation.

Aug. 1—Can walk around comfortably, had a good night, diarrhœa ceased. To continue chloroform as needed.

This woman went her full time, and had a fine healthy child after an easy labor. She was bled again in October for plethora, and used the chloroform almost daily for about five weeks after last report, August 1.

Among the most interesting forms of disease in which chloroform has been used is Convulsions, puerperal, infantile and epileptic. A most interesting case of infantile convulsions, treated by Prof. Simpson, may be found in Braithwaite's Retrospect, part 25, page 71, in which a child twenty-eight days old, used ten ounces of chloroform in 48 hours by inhalation, after which there was no recurrence of the affection. In the puerpural form it is now often used and in the proper cases with excellent results. Epilepsy has not been so frequently treated with it, but when the fits recur rapidly it would seem to offer the prospect of usefulness by suspending the action of the exciting cause. In all this class of cases, it is necessary usually to produce its full effect, the rule being, *to give it only in those cases where there is absence of centric disease.* How far it may be useful when there is disease of the brain or spinal column has not yet been tested, but it would seem as if there might be cases in which it would relieve suffering with safety, and thus prolong life and give more time for the action of other remedies.

5. To procure perfect quiet. This is necessary in some operations where anæsthesia is not needed. The following case is an instance, and will indicate sufficiently well those cases:

CASE.—A lad about six years of age was brought to me with a cherry stone in the external auditory canal. It was in contact with the tympanum, and filled the diameter of the tube. Some attempts had been made at extraction, and the meatus was a little tender; consequently the child would not allow the approach of an instrument. And although he was rolled in a sheet, three stout persons could not holdhim still enough to allow me to act with safety. Chloroform was now given by inhalation until perfect insensibility was induced. The cherry pit was then removed without trouble.

6. As an anti-emetic Chloroform has been inhaled for the purpose of keeping the stomach quiet in cholera, mainly with the view of getting thus the action of other remedies. I can very well suppose that it might be useful in obstinate vomiting from other causes as in pregnancy and in some forms of stomach disease.

The subject of inhalation will not be complete without some remarks on the rules for administering this agent, its effect on the system, and treatment for the asphyxia produced by an over dose.

Rules for its administration: A good article only should be used, one of perfect purity can seldom be had. Chloroform should be once and a half heavier than water, and the fruity odour should not be strong. I am not aware that anything more deleterious than self is ever present even in the worst specimens, the difficulty with such, is, the irritation produced in the air passages, and the failure to induce anæsthesia. The patient being placed in the recumbent position and the operator having ascertained that there is no disease of the heart, brain, or lungs that would unfavorably modify its effects, should pour the fluid on a cloth or handkerchief of small size, and hold it within three inches of the mouth; thus the air will be so freely inhaled with it, that the patient will get about five volumes of air to one of chloroform. And this is near enough to the amount needed for practical purposes and the safety of the subject. Not more than two drachms should be used to begin with for an adult, less proportionally for children. When it has evaporated, which will be within three or four minutes, a short interval should elapse, and if the effect wanted is not produced, the dose should be at once repeated, increased, or diminished, and so on until we attain the desired result. During this procedure, the pulse, eye, and respiration should be carefully watched, the effect produced being the guide and not the quantity used. If the eyes turn up, respiration becomes enfeebled, slow or sterterous, the pulse flags, and the skin cold or wet with perspiration. Then the administration should cease at once. Anæsthesia sufficiently perfect for operating is almost always induced before this condition comes on, and may be known by there being no involuntary winking on touching the eye-lid or eye. In some cases the eyes also turn up in this stage, and there is involuntary contraction of voluntary muscles, but these symptoms alone do not contra indicate the continuance of anæsthesia.

On infants the action of Chloroform is quick, and they throw its effects off rapidly. As age advances, its action is more gradual. Old persons come from under its influence slowly. This dissimilarity between age and childhood is owing to the difference in the activity of circulation and respiration in the two conditions, in both extremes therefore greater caution is necessary in its exhibition.

Chloroform is not forbidden in cases of great debility aud emaciation, or where the system is suffering from the severe "shock" of some great injury, but stimulants should generally be first given in these cases. An empty stomach is desirable, but circumstances will not always allow this, yet if it is so full that it must interfere with respiration in anæsthesia a zinc emetic had better first be given. Food should not be used for some time after the inhalation of chloroform, two or three hours is generally long enough to wait, if taken before vomiting is very apt to follow.

Effects on the system.—When inhaled chloroform acts as a stimulant to the parts frequently exciting cough, &c., this soon subsides, and it speedily enters the circulation and is carried to every part with the blood. This hydro-carbon thus acts directly on the whole cerebro-spinal system destroying its nervous energy and paralysing its functions, as a consequence, sensation, respiration, and the hearts action cease if the dose is sufficient, and death follows from a true asphyxia. Where the chloroform is given more carefully and in less quantity, this overwhelming effect is not seen, the first effect is here often some involuntary motion restlessness or delirium, this generally soon subsides, respiration becomes slower and deeper, pulse slower, voluntary muscles lose their power, eyes turn up and the pupil contracts sluggishly : there is now perfect anæsthesia. If carried further, danger is to be apprehended, and may be known by feeble pulse, respiration slow and feeble, gasping, or stertorous, pupils dilated, and sometimes profuse perspiration and a cold palid surface. "In death from chloroform the respiratory movements cease first, the heart continues to act for some time longer and its then failing is in consequence of the respiratory movements having ceased."

After anæsthesia there is often consciousness long before there is any muscular power or much sensation, and there is sometimes peculiar nervous conditions left so various as not to be described, and lasting for months. Insanity has also resulted but has always so far as recorded cases go, been recovered from in a period of a few weeks.

Post Mortem Appearances.—"After the most powerful doses the lungs are collapsed, the blood natural in color and consistency, the heart placid and empty, or the right side moderately distended by the cava and the left ventricle contracted. The brain is natural. But when death is less instantaneous, the lungs are congested, ecchymosed, emphysematous, the right side of the heart and cava distended sometimes enormously; the sinusses and membranes filled with blood."

Treatment of Chloroform Asphyxia.—The patient if not already recumbent should be laid down in a current of fresh air if possible, and artificial respiration at once commenced and perseveringly kept up. This can always be done from mouth to mouth, moments are not to be lost in waiting for instruments; after each insufflation, pressure must be made on the ribs and abdominal muscles to expel the air; thus imitating the natural act. This plan first recommended by M. Ricord should not be interrupted for any other means, but persisted in at least for fifteen or twenty minutes, even if the hearts action has ceased. An occasional current of galvanic electricity might be passed through the chest, or if the heart does not act then through this organ. Dashing the face and chest with cold water might once or twice be used at the very onset of fatal symptoms. No attempt to give any fluid by the mouth should be made, it will be as likely to run into the lungs as stomach. Some very successful cases of treatment by artificial respiration will be found in Braithwaite's Retrospect, part 28, page 215- 16 and 29. The tongue during this process must be held by an assistant with an instrument, either out of the mouth with a hook or forcess, or kept in its place in the mouth with a spatula. This is very important as the organ is apt to fall back and fill the glottis.

If signs of returning consciousness follow these efforts, dry external warmth should be applied. and as soon as the subject can swallow some warm diffusable stimulant should be given and repeated from time to time until the pulse and respiration are established.

It may be here stated that about fifty cases of fatal asphyxia from chloroform are on record, that there have been double this number never reported, it would be safe to assume.

Aurora, March, 1855.

